# Development of a Quantitative Instrument to Elicit Patient Preferences for Person-Centered Dementia Care Stage 1: A Formative Qualitative Study to Identify Patient Relevant Criteria for Experimental Design of an Analytic Hierarchy Process

**DOI:** 10.3390/ijerph19137629

**Published:** 2022-06-22

**Authors:** Wiebke Mohr, Anika Rädke, Adel Afi, Franka Mühlichen, Moritz Platen, Bernhard Michalowsky, Wolfgang Hoffmann

**Affiliations:** 1 German Center for Neurodegenerative Diseases e.V. (DZNE), Site Rostock/Greifswald, Ellernholzstrasse 1-2, D-17487 Greifswald, Germany; anika.raedke@dzne.de (A.R.); adel.afi@dzne.de (A.A.); franka.muehlichen@dzne.de (F.M.); moritz.platen@dzne.de (M.P.); bernhard.michalowsky@dzne.de (B.M.); wolfgang.hoffmann@uni-greifswald.de (W.H.); 2 Institute for Community Medicine, Section Epidemiology of Health Care and Community Health, University Medicine Greifswald, Ellernholzstrasse 1-2, D-17487 Greifswald, Germany

**Keywords:** patient-centered care, dementia, mild cognitive impairment, patient preferences, patient participation, qualitative research, attributes

## Abstract

**Background:** Person-centered care (PCC) requires knowledge about patient preferences. This formative qualitative study aimed to identify (sub)criteria of PCC for the design of a quantitative, choice-based instrument to elicit patient preferences for person-centered dementia care. **Method:** Interviews were conducted with *n* = 2 dementia care managers, *n* = 10 People living with Dementia (PlwD), and *n* = 3 caregivers (CGs), which followed a semi-structured interview guide including a card game with PCC criteria identified from the literature. Criteria cards were shown to explore the PlwD’s conception. PlwD were asked to rank the cards to identify patient-relevant criteria of PCC. Audios were verbatim-transcribed and analyzed with qualitative content analysis. Card game results were coded on a 10-point-scale, and sums and means for criteria were calculated. **Results:** Six criteria with two sub-criteria emerged from the analysis; social relationships (indirect contact, direct contact), cognitive training (passive, active), organization of care (decentralized structures and no shared decision making, centralized structures and shared decision making), assistance with daily activities (professional, family member), characteristics of care professionals (empathy, education and work experience) and physical activities (alone, group). Dementia-sensitive wording and balance between comprehensibility vs. completeness of the (sub)criteria emerged as additional themes. **Conclusions:** Our formative study provides initial data about patient-relevant criteria of PCC to design a quantitative patient preference instrument. Future research may want to consider the balance between (sub)criteria comprehensibility vs. completeness.

## 1. Introduction

With aging populations, dementia represents a challenge for health care systems worldwide [1]. Globally, around 55 million people have dementia, and there are nearly 10 million new cases every year [2]. The *Global Burden of Disease Study 2019* estimates Alzheimer’s disease and other dementias as the fourth-leading cause of death globally in the age group 75 years and older [3]. Currently, no curative, disease-modifying treatment for all People living with Dementia and Mild Cognitive Impairment [hereinafter commonly referred to as “PlwD”] exists. PlwD need a timely differential diagnosis as well as evidence-based treatment and care, which ensures a high Quality of Life (QoL) [1,4].

Person-centered care (PCC) is the underlying philosophy of the Alzheimer’s Association Dementia Care Practice Recommendations. A person-centered focus is viewed as the core of quality care in dementia [5]. Many countries include a PCC approach in their national guidelines and dementia plans [6,7,8,9,10,11,12]. It follows a non-pharmacological, sociopsychological treatment approach and challenges the traditional clinician-centered or disease-focused medical model to instead suggest a model of care, which is customized to each person [13]. This customization requires knowledge about the recipient’s needs and preferences [14,15]. Among PlwD, some research about preferences exists, however, little is known about preferences elicited through quantitative, in particular, choice-based preference methods [16,17]. A recent literature review focused on *decision-making tools* with PlwD by Ho et al. [18] found that earlier studies often applied qualitative methods and Likert-type scales. Harrison Dening et al. [19] elicited preferences from dyads during qualitative interviews, van Haitsma et al. developed an extensive Likert-scale based *Preferences for Everyday Living Inventory (PELI)* for elicitation of preferences in community-dwelling aged adults [20]. These methods, however, fall short in *quantifying*, *weighing* and *ranking* patient-relevant elements of care to measure their relative importance and identify most/least preferred choices. Such information can be assessed with quantitative, choice-based preference measurement techniques from multi-criteria decision analysis (MCDA) [21]. Groenewoud et al. [22] addressed relevant aspects of outpatient care and support services for people with Alzheimer’s disease by application of a quantitative, choice-based preference instrument (Discrete Choice Experiment (DCE)), which, however, was carried out with patient representatives and not the patients themselves. Other MCDA techniques commonly used in health care include best–worse scaling (BWS) [23] and the Analytic Hierarchy Process (AHP) [24]. The AHP, depending on the number of elements included, may require to ask many questions. DCEs, depending on the number of choice sets included (full vs. fractional factorial design), usually include fewer but cognitively more challenging questions. BWS distinguishes between three basic cases; object scaling (case 1), attribute or profile scaling (case 2) and multi-profiling (case 3), each case including various experimental designs, number of choice sets and questions. Hence, in BWS, the cognitive demands of included questions increase with each case [23]. To elicit patient preferences from people with cognitive impairments, the AHP has been suggested, as it may be more feasible than other MCDA techniques due to simple pairwise comparisons with only two individual aspects of a complex decision problem [25]. To keep the number of choice questions doable, the number of elements to include in the AHP model needs to be considered in the early development stages.

MCDA techniques, including the AHP, comprise the development of attribute/criteria-based experimental decision models for preference measurement [26,27]. The validity of an attribute/criteria-based experiment depends on the researcher’s ability to appropriately identify and specify the included criteria [24,26,27,28]. Poorly identified criteria can have negative implications for the design and conduct of AHP surveys and increase the risk of inaccurate results, which in turn can misinform potential policy implementation. The risk of bias, i.e., researcher bias, in quantitative preference measurement studies can be reduced by a rigorous, systematic, and transparently reported identification of (sub)criteria [28,29]. Several methods have been suggested for AHP development, e.g., literature reviews, existing conceptual and policy-relevant outcome measures, theoretical arguments, expert opinion reviews, professional recommendations, patient surveys, nominal group ranking techniques and qualitative research methods [24]. Coast et al. [30] emphasize the limitation of attribute and level derivation only from a review of the literature and suggest the additional application of qualitative methods for attribute elicitation. These methods include the right instruments to capture and reflect the perspective and experiences of the decision makers. Only accurately described *formative* qualitative studies applied to derive (sub)criteria give readers the opportunity to judge the quality of the resulting decision model for preference elicitation [29]. Despite a recent increase in publications about pertinent studies, there is still a lack of both evidence and experience.

To the best of our knowledge, no previous research has reported the qualitative identification of patient-relevant (sub)criteria of PCC among community-dwelling PlwD. Our study aimed to fill this gap with this rigorous process report on (sub)criteria identification for the design of a quantitative, choice-based instrument, an AHP, to elicit patient preferences for PCC among community-dwelling PlwD.

## 2. Methods

We followed the guidelines for reporting *formative* qualitative research to support the development of quantitative preference survey instruments by Hollin et al. [29].

### 2.1. Qualitative Approach

We applied a narrative qualitative approach to cover the PlwD’s individual experiences [31]. As this study employed a flexible strategy, characterized by the inclusion of life histories and interpretive analysis, the research paradigm followed critical realism [32].

### 2.2. Theoretical Framework

**Figure 1:**
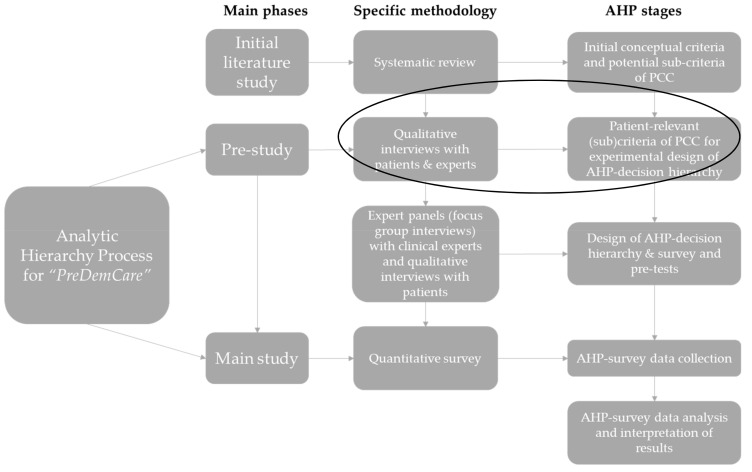
The mixed-methods design of the AHP for *PreDemCare* (own illustration inspired by [28]). Note: The initial literature study refers to a previously conducted systematic review [36]. AHP survey data will be analyzed with the principal right eigenvector method following Saaty [37]. Abbreviations: AHP = Analytic Hierarchy Process, PCC = person-centered care.

The overarching AHP-study, *“PreDemCare”* [33,34] adopts a sequential mixed-methods design for final instrument development [35], depicted in Figure 1. For the pre-study phase, we followed a qualitative design informed by a previous systematic review to identify relevant (sub)criteria, which would serve the development of an AHP. This report focuses exclusively on the pre-study phase of the overarching AHP study and describes the first qualitative component in detail.

#### 2.2.1. Theoretical Perspective

The theoretical perspective behind the overarching *PreDemCare* study, including this formative qualitative study, is guided by the theoretical foundations of the AHP, cf. Mühlbacher and Kaczynski [24]. The AHP is a method of prescriptive or normative decision theory, which provides the decision maker with techniques to reach a meaningful and plausible/rational decision [24,38]. The decision maker solves the decision problem based on predefined decision goal criteria and individual or group-specific priorities to identify the use-maximizing alternative systematically.

#### 2.2.2. Initial Systematic Literature Review

The process of (sub)criteria identification [24] was based on a systematic review, which aimed to identify key intervention categories of PCC for PlwD. The results can be reviewed elsewhere [36]. Nine key components of PCC for PlwD were identified: *Social contact, physical activities, cognitive training, sensory enhancement, daily living assistance, life history-oriented emotional support, training and support for professional caregivers, environmental adjustments, and care organization.* Based on these findings from the literature, a comprehensive list of conceptual (sub)criteria was derived, depicted in Table 1.

The qualitative pre-study entailed (1) an expert panel with internal dementia-specific qualified nurses, so-called *Dementia Care Managers* (DCMs) [39] and (2) patient interviews with community-dwelling PlwD and informal caregivers (CGs) as silent supporters who live in diverse regions in rural German Mecklenburg-Western Pomerania.

### 2.3. Researcher Characteristics and Reflexivity

The authors WM and AR, public health scientists with qualitative research experience, conducted the interviews. AR has many years of quantitative patient preference research experience [24,40]. If one interviewer was hindered to participate, an experienced DCM from the site took over this role. Study nurses in ongoing clinical trials at the site (ClinicalTrials.gov identifiers: NCT04741932, NCT01401582, German Clinical Trials Register Reference No.: DRKS00025074) functioned as gatekeepers to access the PlwD for patient interviews, as they may be perceived as trustworthy by the participants. None of the PlwD and informal CGs interviewed knew the scientists beforehand but were aware of their professional roles.

### 2.4. Sampling Strategy and Process

For the expert panel, two of the most experienced DCMs were selected at the site. PlwD for the patient interviews were selected by typical case sampling [41,42], a type of purposive sampling [43], from ongoing clinical trials at the site. The gatekeepers emphasized the independence of this study from the ongoing clinical trials. Informal CGs were invited to join as silent supporters.

### 2.5. Sampling Adequacy

For the determination of sampling adequacy in a *formative* qualitative study, such as ours, to support the development of a quantitative preference instrument, we oriented ourselves in recently published recommendations by Hollin et al. [29]. Following these, the focus should not be the number of subjects, which may differ from *general* qualitative research, but the strategical collection of actionable input for the development process. The latter includes the requirement of diversity in perspectives.

We addressed the diversity of perspectives by the inclusion of different stakeholders. Additionally, the initial overall sample size for the patient interviews *n* = 10 was informed by the expected saturation point [43] based on experiences from previous formative qualitative research for the development of quantitative preference instruments [67,68,69,70,71,72] and expected restricted access to PlwD due to the COVID-19 pandemic. The latter included ethical reflections in the study team to limit the risk associated with contact for both the vulnerable patient group and team members.

The identified (sub)criteria were subsequently revisited and assessed again during pre-tests of the to-be-developed AHP survey instrument in two expert panels with *n* = 4 DCMs, *n* = 4 physicians and *n* = 11 PlwD, cf. Figure 1. However, details on this subsequent stage in instrument development for the *PreDemCare*-study [34] lie outside the scope of this report.

### 2.6. Sample

The expert panel included *n* = 2 DCMs from the site’s staff. Patient interviews included *n* = 10 PlwD and *n* = 3 informal CGs (mainly as silent supporters).

### 2.7. Ethical Review

The overarching preference study *PreDemCare*, including this pre-study, was evaluated and approved by the Ethics Committee at the University Medicine Greifswald (Ref.-No.: BB018-21, BB018-21a, BB018-21b).

### 2.8. Data Collection Methods, Sources and Instruments

WM conducted the expert interview via video conference software. After translation to German, the DCMs reviewed the literature-derived conceptual criteria and their descriptions, as well as the sub-criteria, including respective icons for comprehensibility, and made suggestions for improvement. The expert interview was not recorded or transcribed. Data were collected with field notes. Changes were implemented immediately. The expert-reviewed material was prepared for the subsequent patient interviews.

Subsequently, individual narrative interviews [43] were conducted with PlwD in their homes or in day-care centers over the time period April–May 2021. All interviews were conducted in adherence to a strict hygiene protocol developed at the site during the COVID-19 pandemic. Method and setting were chosen to consider the vulnerability of this population appropriately. To ensure a comfortable and non-stressful interview situation, PlwD could invite their informal CGs to support them during interviews. It was, however, emphasized that the informal CGs should not act as proxies and answer the majority of the questions on behalf of the PlwD. WM conducted the interviews, while a second interviewer (AR or a DCM) took field notes. All interviews were recorded. All participants were informed about the purpose and content of the study, i.e., to obtain their opinion about relevant criteria of individualized homecare via the interview, including a card game, which would be used in research for the subsequent development of a survey. The interviewers explicitly stated that no tests would be performed. The audio tape was started after the introduction of the participants to ensure privacy. The average interview time was 60 min.

We used a self-developed semi-structured interview guide, oriented in Danner et al. [73], to ensure an efficient structure of the interview and simultaneously give the participants room to elaborate freely. Oriented in Danner et al. [25], we repeated after each pairwise comparison during the card games what the patient said with his/her judgement, e.g., *“With your judgement you are saying that [Criterion X] is very much more important to you than [Criterion Y]; is this what you wanted to express?”*, to make sure the information and tradeoffs presented during the card games were understood. We included an initial self-developed sociodemographic questionnaire for patient characteristics. Time since diagnosis and severity of cognitive impairment was determined during recruitment based on inclusion criteria (indication of MCI or early to moderate staged dementia) by the internal study nurses as gatekeepers based on their most recent assessment with a validated instrument in the respective clinical trial (Mini Mental Status Test (MMST)) [74] and/or Structured Interview for the Diagnosis of Dementia of the Alzheimer Type, multi-infarct dementia and dementias of other etiology according to DSM-III-R and ICD-10 score (SISCO) [75]).

The literature-based and expert-reviewed conceptual (sub)criteria were printed on cards in A5 format. Oriented in Danner et al. [73], criteria cards were presented to the PlwD as part of three card games to identify the most important and patient-relevant criteria of PCC. Card game 1 included sorting the criteria cards on three stacks (important, neutral, not important). Card game 2 included sorting the important criteria cards from card game 1 on two stacks (very important, less important). Results from the final ranking game, which included sorting the very important criteria cards from card game 2 in ranking order, were numbered according to their position awarded in this ranking. All results were documented with photographs and field notes. Blank cards were kept aside in case the PlwD mentioned additional criteria that had not been identified from the literature or in the expert interviews. Sub-criteria cards were only presented if there was time and energy left. If so, we asked about the appropriateness of the sub-criteria, their wording and the graphical design of included visual aids (ICONs).

By the described utilization of diverse data collection methods and different observers, we ensured both data and investigator triangulation [43].

### 2.9. Data Processing and Analysis

#### 2.9.1. Card Games

Card game results were transferred into Microsoft^®^Excel2019 for a comprehensive overview. Ranking results were coded on a 10-point scale (rank 1 = 10 points, rank 2 = 9 points and so forth; excluded criteria were assigned zero points), whereupon sums and means for criteria across interviews were calculated.

#### 2.9.2. Audio Recordings

Audio recordings were transcribed verbatim by WM. If names had been mentioned during the interview, these were not transcribed but replaced with, e.g., “XXX”, to ensure privacy. Two reviewers, WM and AA, coded transcripts line by line with qualitative content analysis [76,77,78] in Microsoft^®^ Word2019. Oriented in Hshie & Shannon [78], we used elements from both conventional and directed qualitative content analysis, i.e., deductive analysis was guided by the interview guide and focused on information necessary to collect, cf. categories 1.–5. in Supplementary Material Codebook S1, but inductively other observations made were allowed to arise as additional categories from the transcripts, cf. category 6 in the Codebook S1. Concretely, each reviewer coded the first interview independently based on the interview guide and the conceptual criteria identified from the literature, cf. Table 1, but allowed for new categories to emerge. Subsequently, the reviewers discussed their codes and categories and agreed on a codebook. The codebook was revisited after independent coding of the second interview, and the strategy suggested was confirmed by both reviewers. Each reviewer coded the remaining interviews (*n* = 8) independently.

For categorization of the coded meaning units, coded transcripts from both reviewers were printed. Coded meaning units were discussed by both reviewers, cut out and assigned a tracker (interview number_lines in transcript). By this, we could trace back the distinct coded section and review it in its context, if necessary. Meaning units were hence sorted into the categories as given by the matrix from the Codebook S1.

Transcript and card game analyses were discussed in a final meeting between all authors until consensus on categorization was achieved. The finally categorized meaning units were transferred into digital format with Microsoft^®^Word2019.

## 3. Results

Patient characteristics are depicted in Table 2.

Six categories emerged from the analysis of the material: (1) patient-relevant criteria of PCC, (2) new criteria of PCC from the patient’s perspective, (3) plausible sub-criteria, (4) overlapping of criteria, (5) wording and comprehensibility and (6) other observations; (6a) reactions by patient, (6b) interaction with informal CG, (6c) explorative vs. ranking card game, (6d) setting and (6e) COVID-19.

### 3.1. Patient-Relevant Criteria

PlwD had preferences, and by use of the sorting and ranking card game, PlwD were able to express their preferences. Table 3 presents the list of criteria as identified after an analysis of the ranking card game. Six criteria were chosen for final inclusion in the AHP decision model and survey; social relationships, cognitive training, organization of health care, assistance with daily activities, characteristics of professional caregivers and physical activities.

### 3.2. New Criteria of PCC

All PlwD were asked whether we had missed criteria of PCC, which were important to them and not included in the criteria presented by us. No PlwD gave an indication of new criteria necessary to include, cf. Table 4. Hence, the literature-derived criteria were confirmed while reduced to a doable amount of criteria for the design of the AHP decision model and survey.

### 3.3. Plausible Sub-Criteria

Based on our observations during the patient interviews, where most participants got tired after ~60 min before we could show the sub-criteria cards, we decided that the AHP decision model and survey had to be kept as simple and short as possible. To limit the pairwise comparisons and to reduce the length and complexity of the planned survey, we decided to elicit and include only two sub-criteria per criterion in the AHP decision model, based on the PlwD’s initial elaborations about the presented criteria cards. Plausible sub-criteria are depicted in column four in Table 3.

### 3.4. Overlapping of Criteria

The participant’s elaborations about the cards gave indications about the potential overlap of criteria, cf. Table 4. Consequently, we decided to merge literature-derived criteria 1 *(Social Activities)* and 6 *(Support with worries)*, as well as criteria 8 *(Information for informal CGs)* and 10 *(Organization of care)*, cf. Table 1, which resulted in the criteria *“social relationships”* and *“organization of health care”,* cf. Table 3.

### 3.5. Wording and Comprehensibility

The participants had difficulties with the criteria’s general formulations, cf. Table 4. Once provided with concrete examples, the participants could relate well to the criteria. We decided to delete extensive criteria descriptions and instead described them with examples from the participant’s elaborations, cf. Table 3, column two.

Dementia is a sensitive topic. To prevent discontinuation of interviews, we had to adapt dementia-related terms in the interview guide and the card game. Consequently, the final (sub)criteria in Table 3 avoid dementia-related wording.

### 3.6. Other Observations

Several inductive observations emerged from data analysis, as presented in the following.

#### 3.6.1. Reactions by PlwD

Initially, some participants were nervous, as some expected a test and wanted to “perform well”, despite explicit explanations by the interviewers that only their opinion was important to inform the subsequent development of a survey and no test would be performed. Some participants had difficulties dealing with “dementia” as a topic. During interviews with informal CGs or a DCM as a second interviewer present, some participants were “keen to please”.

#### 3.6.2. Interaction with Informal CGs

During three interviews, informal CGs joined the PlwD. Some PlwD displayed concern about losing their informal CG, cf. Table 4. The relationship between PlwD and CG was at times affected by the better fitness of the CGs, who could be overstepping. During elaborations about help with, e.g., daily activities, particularly male PlwD showed expectations that their wife would take care of this.

#### 3.6.3. Explorative vs. Card-Game Responses

Some PlwD had difficulties with the initial explorative part, which required abstract thinking to elaborate on the presented criteria and related experiences and wishes, cf. Table 4. The subsequent card game, which included concrete comparisons and sorting of the cards, did not pose a problem for the PlwD.

Many PlwD were still physically fit and did not need help with daily activities or adjustments to the living environment. Some elaborated “imagine if…” thoughts, i.e., if they would require help in the future would they be happy to receive it and how they would want to receive it. Others did not want to think about the unknown future and could not elaborate on what they would wish for their care, cf. Table 4.

#### 3.6.4. Setting

The interviews were conducted in the German Federal State Mecklenburg-Western Pomerania, a former part of the German Democratic Republic (GDR). Elaborations about certain criteria, e.g., criterion 10, cf. Table 1 were associated with examples related to this setting. These examples from the past political and economic systems helped with the PlwDs’ understanding of the criteria, cf. Table 4. Consequently, we decided to include these examples (e.g., polyclinics in the GDR) to describe the patient relevant criteria and sub-criteria, cf. Table 3.

#### 3.6.5. COVID-19

The PlwD’s elaborations were affected by COVID-19, cf. Table 4. Especially the criteria “access to social activities” and “physical activities” were mentioned as impacted by the COVID-19 restrictions.

## 4. Discussion

Our article contributes to the limited literature with a report on the systematic process of initial (sub)criteria derivation for the development of an AHP decision hierarchy and survey to elicit patient preferences for PCC among community-dwelling PlwD. This *formative*, qualitative research study was built on the previous identification of conceptual (sub)criteria by a systematic literature review. PlwD had preferences, and by use of the card game, they were able to express their preferences. The analysis resulted in six patient-relevant criteria, each with two sub-criteria; *social relationships (indirect contact, direct contact), cognitive training (passive, active), organization of care (decentralized structures & no shared decision-making, centralized structures and shared decision making), assistance with daily activities (professional, family member), characteristics of professional CG (empathy, education and work experience) and physical activities (alone, group)*. No further criteria emerged from the interviews. Overlapping criteria were merged. The wording had to be substantially simplified by deletion of extensive criteria descriptions and replacement with concrete examples, and adjusted to dementia-sensitive language. Some PlwD initially were nervous to “perform well”, as they expected to be tested despite explicit explanations by the interviewers that this was not the case. COVID-19 was a present topic during the participants’ elaborations.

The initial systematic review allowed us to identify a preliminary broad set of possibly patient-relevant (sub)criteria. Key quotations presented in Table 3 give a clear indication that the selection of (sub)criteria was rooted in and supported by the voices of the decision makers. Furthermore, this qualitative pre-study gave us the opportunity to identify and exclude overlapping criteria in compliance with the credibility criteria of an AHP decision model [24].

Three of the identified six criteria—social relationships, cognitive training and assistance with daily activities—reflect attributes used in a previous quantitative, choice-based preference study with PlwD and their informal CGs [46]. We had oriented ourselves in Chester et al. [46] for the derivation of conceptual sub-criteria prior to the interviews, cf. Table 1. However, Chester et al. [46] applied another MCDA technique, a DCE, and included both PlwD and their informal CGs as respondents.

If we had relied only on results from the initial systematic review [36] and Chester et al. [46], the final list of (sub)criteria and the resulting number of pairwise comparisons would have become too extensive for this patient group. Furthermore, we would not have known if all identified criteria were relevant and important from the patient’s perspective. Hence, we tested if the criteria from the literature review were patient-relevant in terms of future decision making. This underlines the importance and necessity of conducting *formative* qualitative studies for contextual and population-specific appropriateness of the AHP (sub)criteria [24,26,27,30].

Despite explicit explanations by the interviewers, some PlwD were initially nervous to “perform well” as they expected a test. This reaction may be based on experiences with assessments for cognitive impairment in the clinical trials which we had recruited from. It may also be that the participants tried to hide their cognitive impairment due to the associated stigma with the diseases, as found by Xanthopoulou & McCabe [79], and hence wanted to perform well during the interview. Future quantitative preference research with PlwD may want to pay particular attention to avoiding expected or perceived test situations and preparation, respectively.

Corona (COVID-19) was a present topic during the participants’ elaborations, especially concerning access to social and physical activities. Lack of access to services and support due to COVID-19-related lockdowns has only recently been raised as a topic of great concern for this patient group [80,81]. It may be that the importance of criteria was affected by the COVID-19 measures, i.e., that the criteria’s relative importance was affected by current unmet needs. However, preferences are based on the processing of needs, values and goals and may shift as the social environment or contextual circumstances change [82]. It might also be that the COVID-19 measures simply enforced existing preferences for PCC criteria among PlwD. This phenomenon could be examined further by future research.

Even though potential clinical implications of our findings based on a small sample size are limited, the identified (sub)criteria of PCC serve the development of an AHP survey, which hence shall be used to elicit patient preferences for person-centered dementia care on a larger scale. Van Til and Ijzerman highlighted the advantage of quantitative preference elicitation methods for measurement of patient preferences on a larger and representative scale, which in turn would allow for reflection of the patient perspective in regulatory/health policy decisions [83]. As indicated by Mühlbacher [21], knowledge about most/least preferred health care options may help to increase acceptance and adherence to interventions among patients. Prioritization in the provision of those interventions accepted and preferred and avoidance of those options less preferred may reduce the financial pressure on health care systems [21]. This may affect both routine care and new concepts of care [40]. PCC requires knowledge about patient preferences [14,15,20,84]. Furthermore, *Shared Decision Making* between the health care provider and the patient is a core element of PCC [36,85]. PlwD as patients are “experts by experience”—hence, incorporation of their perspective in care decision making is of importance. Jayadevappa et al. [86], who applied a quantitative, choice-based preference instrument, saw i.a. improved satisfaction with care and decision, as well as reduced regrets. Quantitative preference elicitation instruments, such as the AHP, may form a powerful instrument for consideration of the patient perspective in dementia care decision making on a larger scale [83]. However, the validity of quantitative, criteria-based preference elicitation instruments depends on appropriate identification of the included criteria to reduce the risk of bias and inaccurate results [24,26,27,28]. The latter can be reduced by a rigorous, systematic and transparently reported identification of (sub)criteria [28,29], as in this current study, which provides initial data of patient-relevant (sub)criteria for the design of an AHP decision hierarchy and survey for person-centered dementia care.

### Limitations

Our study has several limitations. Conceptual (sub)criteria identified from literature had to be translated from English to German. Information could have been lost in translation or content compromised by language errors. However, the translation by WM was reviewed by the other authors, as well as by the DCMs during the expert panel, which mitigated the probability of possible translation flaws. The expert panel included a small number of participants (*n* = 2), who were internal colleagues of members of the study team. However, expert perspectives were not the primary objective of this pre-study. Consultation with clinical experts can, nonetheless, provide the basis for identifying the full set of (sub)criteria for subsequent qualitative research with patients and is in accordance with good research practices in patient preference research [87]. Similar to our study, Kløjgaard et al. [88] only included *n* = 2 experts in the formative qualitative study phase for the development of the quantitative preference instrument. Compared to usual sample sizes in general qualitative research, the number of participants during the patient interviews may appear low. As aforementioned, cf. Section 2.5, we oriented ourselves in a recent publication by Hollin et al. [29], which entailed guidelines for *formative* qualitative research, such as ours, to support the development of quantitative preference instruments. The authors emphasize that sampling in these study phases should not focus on the number of units but on collecting actionable input for the development process, which needs a diversity of perspectives. They underline that sampling adequacy in *formative* qualitative research may entail smaller samples than in *general* qualitative work, which given the limited study purpose, may be adequate [29]. To complement suggestions by Hollin et al. [29] and inform the expected saturation point as guidance for sample size determination, we oriented ourselves in previous quantitative patient preference research, including works by second author AR, which report similar sample sizes in the *formative* pre-study phase(s) [67,68,69,70,71,72]. In this formative qualitative study, saturation started to appear from patient interview number six. The remaining four interviews clarified and consolidated the ranking of criteria, especially of “social relationships”, “cognitive training”, and “physical activities”. By the inclusion of several stakeholders, we ensured a diversity of perspectives. We could have conducted focus group interviews with the PlwD as Danner et al. [73]. However, due to the sensitivity of the topic, the vulnerability of the patient group, and COVID-19-related restrictions on group meetings, we refrained from this option. Another option might have been to administer the card game as an online patient survey for the identification of patient-relevant (sub)criteria [24], by which risks associated with contact during the COVID-19 pandemic would have been limited, and the sample size potentially could have been increased. However, an online patient survey without interviewer assistance with this particular patient group—aged adults with cognitive impairments, oftentimes living in rural areas, which may have limited access to the internet and a lack of necessary digital literacy [89]—was deemed not feasible by the study team based on previous research [25] and experiences from other projects at the site [90]. As criteria-related questions and card games took longer than expected and most PlwD got tired, we could not show the sub-criteria cards and ask for feedback on their appropriateness and comprehensibility. Instead, we elicited plausible sub-criteria from the participants’ initial elaborations about the criteria cards, which, together with the designed ICONs, were planned to be tested for their appropriateness during the subsequent pretests of the AHP survey, cf. Figure 1. Generally, interviewers should not guide interviewees and rather aim for open interview questions [91]. This requirement was difficult to fulfill with this patient group and research aim. PlwD had difficulties with open/abstract questions and needed guidance throughout the interviews with concrete questions to create a comfortable interview situation, as observed in previous research [92]. Future patient preference research with a cognitively impaired population may want to consider these observations. For some PlwD, elaborations about selected criteria required imagination of potential scenarios in the future. This resulted in some inconsistency between the explorative part and card games, which could be an early indication of a known methodological problem with the AHP. Thus, the AHP is criticized for the mere pairwise comparisons not fully reflecting to a real decision-making situation, as the decision maker never is confronted with the entirety of a decision problem but only with individual aspects of an overall decision [93]. It could also be an indicator that the cognitively impaired patient group did not understand the information and tradeoffs presented during the card games. However, we followed the same approach as Danner et al. [25], i.e., to repeat after each pairwise comparison during the card games what the patient said with his/her judgement, to counteract this potential problem. Per our observations, cf. Section 3.1 and Section 3.6.3, the patients understood the information and tradeoffs presented during the card games well, compared to the more explorative part at the beginning of the interviews. Hence, we are confident in the results of the presented tradeoffs. As we remained compliant with our research focus and collected a manageable amount of data in a short period of time, the requirements for credibility and dependability with regard to the study’s trustworthiness were viewed as fulfilled [94]. Transferability of findings is limited due to the aforementioned rather small sample sizes of included subjects, the specificities of our setting and related cultural differences. Nevertheless, due to the rigor of the methodological process and reporting, we consider our findings trustworthy.

## 5. Conclusions

This formative qualitative study complements the limited literature with initial data about patient-relevant criteria of PCC for PlwD to design a quantitative preference instrument. To the best of our knowledge, our research is among the first to provide insight into the methodological processes of (sub)criteria development for the subsequent design of an AHP for a cognitively impaired population. PlwD had preferences and, by use of the card game, were able to express their preferences. The transferability of our findings is limited due to the comparatively small sample sizes of included subjects. Aside from the consideration of larger sample sizes, future research should pay particular attention (a) to clarify the purpose of the study and to ensure tradeoffs are understood by the participants, (b) to include simple and concrete rather than abstract as well as dementia-sensitive wording and (c) to account for the energy required in relation to the age and cognitive status of the participants, as well as challenges in qualitative research with this population, which requires great researcher flexibility. A consideration of our observations in future quantitative preference research with PlwD may help to increase the confidence in such research.

## Figures and Tables

**Table 1:** Conceptual criteria and potential sub-criteria oriented in systematic literature review [36].

*Function (Descriptions Oriented in [44,45])*	*Oriented in named intervention categories by Dickson et al. [44] & Clarkson et al. [45], as well as attributes and levels defined in a previous Discrete Choice Experiment by Chester et al. [46]*	
	*Potential criteria (oriented in intervention categories to provide Person-Centered Dementia Care as identified in Mohr et al. [36])*	*Plausible sub-criteria (oriented in provider, format, setting and/or intensity as identified in Mohr et al. [36])*
To provide access to different forms of social contact to counterbalance the limited contact with others that may be characteristic of the experience of dementia. This social contact may be real or simulated [45]. **Examples of activities [47,48,49,50,51,52]:** Social simulation tool (e.g., robotic animal, lifelike baby doll, baby video, respite video, stuffed animal, family pictures and family video, writing letters), one-on-one interaction (incl. active listening and communication), conversation (e.g., general and based on newspaper stories, pictures, etc.), group activity	**(1) Possibilities for social activities**	Difficult to access Group activities, e.g., in the local community house 1-to−1 contact at home with family member/professional CG/volunteers
To provide structured activities and/or exercise to provide meaningful and engaging experiences that can be a useful counterbalance to difficult behaviors [45]. **Examples of activities [47,49,52,53,54]:** outdoor walks, gardening.	**(2) Possibilities for physical activities**	Difficult to access Group activities, e.g., in fitness studio Individual activities with a personalized trainer at home
To provide enhancement and stimulation of cognitive functions through guided practice on a set of standard tasks, reflecting memory, attention or problem solving [45]. **Examples of activities [47,48,49,51,52,53,54,55]:** puzzles and games, reading, poetry, theatre, arts and crafts, work-like activities, housekeeping tasks, videos and television, sorting.	**(3) Cognitive training**	Difficult to access Activities outside the home, e.g., in memory clinic Activities at home with family member/speech therapist/ergo therapist/volunteers
To increase or relax the overall level of sensory stimulation in the environment to counterbalance the negative impact of sensory deprivation/stimulation common in dementia [45]. **Examples of activities [47,48,49,51,52,53,54,56]:** music (e.g., listening, singing along, including in conversations and care), sensory stimulation with different materials, e.g., hand massage with lotion, smelling fresh flowers, preferably in a white and quiet room (refers to Snoezelen).	**(4) Activities for sensory stimulation or relaxation**	Difficult to access Activities to access outside home, e.g., in physiotherapy- and massage clinic Activities at home with physio therapist/masseur/music therapist
To assist with basic care, e.g., provision of laundry services, basic nutrition and help with activities of daily living [45]. **Examples of activities [47,49,54,55,57]:** care (e.g., help with personal hygiene and dressing, discussions about health status with physician), food or drinks, person-centered showering/towel bath.	**(5) Help with activities of daily living**	Rarely available Three times per week with educated staff and consistent staffing Once per day with educated staff, but changing staff
To address feelings and emotional needs through prompts, discussion or by stimulating memories and enabling the person to share their experiences and life stories; undertaken to counterbalance and help people manage difficult feelings and emotions [45]. **Examples of activities [47,48,50,54,58,59,60,61,62]:** telling life histories, work with reminiscence and self-validation.	**(6) Attention and support with worries, feelings and memories**	Rarely available Accessible via a telephone hotline Through specifically educated advisor/priest/professional CG/family member
To change interactions between CGs and PlwD, including: psycho-education; integrated family support, such as counseling and advocacy; training in awareness and problem solving; and support groups [44]. **Examples of activities [47,48,50,51,52,55,56,58,59,60,61,62,63,64]:** training, further education and counseling of professional caregivers (e.g., about dementia-related medication), work experience	**(7) Dementia- and PCC specialized training for professional CGs _a_**	CG assistant with three years of work experience Examined professional CG with 1.5 years of work experience Examined professional CG with additional certifications and half a year of work experience
Provision and access of information about dementia, as well as PCC for informal CGs. Emotional support of informal CGs. Inclusion of the family in care decisions. **Examples of activities [47,48,50,51,52,55,56,58,59,60,61,62,63,64]:** access to informational material via GP, Dementia support groups or the internet, self-help groups for informal caregivers, inclusion in care decisions by professional CG and/or GP.	**(8) Dementia focused information and support for family CGs _a_**	Difficult to receive Easy to receive Very easy to receive
To modify the living environment, including the visual environment, in order to lessen agitation and/or to wander and promote safety [45]. **Examples of activities [47,50,55,63]:** Physical aids, homey adaptions to environment, assistive technology, sign-age, reduction of noise and clutter.	**(9) Adjustments of the environment**	Not accessible In one room, e.g., the bathroom In the complete living area
To connect and bring together different services around the person; to advise on and negotiate the delivery of services from multiple providers on behalf of the person to provide benefit [45]. **Examples of activities [47,50,51,52,55,58,60,61,63,64,65]:** shared decision- making between professional CG and/or GP and PlwD, interdisciplinary and integrated care planning incl. consistent staffing, case management, special dementia units in hospitals.	**(10) Organization of care**	No shared decision making and integrated health services Some shared decision making and integrated health services Always shared decision making and integrated health services
Possible additional out-of-pocket payments.	**(11) Additional cost _b_**	20 € per month (240 € per year) 40 € per month (480 € per year) 80 € per month (960 € per year)
Possible additional waiting time, which would have to be taken into account for certain offers.	**(12) Waiting time _b_**	11–14 days 7–10 days 3–6 days

Abbreviations: CG = Caregiver, GP = General Practitioner, PlwD = Person living with Dementia. _a_ Initially, these criteria were one intervention category in the systematic review. To avoid too long criteria labels, we decided to split this category into two potential criteria—one focused on professional caregivers, one focused on informal caregivers. _b_ The cost and waiting time criteria were added to the conceptual criteria from the literature, as these are common criteria in other quantitative preference research studies [66].

**Table 2:** Patient Characteristics (*n* = 10).

Characteristic		*n*
Age		
	60–71	2
	71–80	2
	81–90	4
	>90	2
Gender		
	Female	4
	Male	6
Family status		
	Married	5
	Widowed	3
	Divorced or separated	2
Highest educational degree		
	No degree	1
	8th/9th grade	4
	10th grade	2
	Degree from a technical/vocational college	1
	Degree from a university of applied sciences or university	2
Monthly net income		
	500–1000 €	2
	1001–1500 €	2
	1501–2000 €	1
	Prefer not to say	5
Time since diagnosis of dementia _a_		
	1–2 years	3
	2–5 years	3
	More than 5 years	3
	Not known	1
Stage of cognitive impairment _b_		
	Early	8
	Moderate	2
Subjective assessment of current health status		
	Good	4
	Satisfactory	5
	Less good	1

_a_ Determined by study nurses during most recent visit in clinical trial the participant had been recruited from. _b_ Determined by study nurses based on most recent assessment with validated instrument (MMST [74] and/or SISCO [75]) during most recent visit in clinical trial.

**Table 3:** Derivation of list of AHP criteria and plausible sub-criteria (ordered from most preferred to least per card game results).

Criterion	Examples _a_	Key Quotations from Qualitative Data (Individual Interviews with *n* = 10 PlwD and *n* = 3 Informal CGs)	Plausible Sub-Criteria	Final Inclusion
Social relationships	Conversations, writing letters, phone calls, meeting friends, club room in facility of community housing, attention and support with worries and feelings	*P: So. That you are in touch with other people. That you don’t say no to the connection to other people, but that you look for it [the connection].* (Int9, lls. 14–15) *I1: Do you prefer direct contact with people?* *P: Yes yes.* *I1: Ok. How about a phone call?* *P: Well I can make a phone call, but I mostly avoid it.* *I1: Because you prefer direct contact?* *P: Yes.* (Int9, lls. 24–29)	Indirect contact, e.g., phone calls, writing letters Direct contact with people	Yes
Cognitive training	Listening to the radio, crossword puzzles, puzzles and games, reading the newspaper, reading books, theater, arts and crafts, work-related tasks, watching TV, cleaning.	*P: News. All the news I can get. Or comments. So the radio is important to me. I’ve already bought a portable radio like this. So I was looking for the smallest and that was the smallest. Smaller was not possible. And that’s important to me.* (Int7, lls. 155–157) *P: Yeah…I do that…well play… we used to play Skat [German card-game] too. […] But now…because of Corona…we always played Skat on Sundays and then it was also the afternoon of games… we had an afternoon where we sat and talked at a long table…* (Int2, lls. 93–97)	Passive, e.g., watching TV, listen to the radio Active, e.g., crossword puzzles, reading, games	Yes
Organization of health care	*See sub-criteria.*	*I1: […] Polyclinics. You surely know them from the GDR, where everything was under one roof. [...]* *P: Hmm, we still have that in the medical center.* *I1: Hm, do you think that’s good?* *P: I think that’s good. That is still like before. [...]* *I1: And would it be important to you that it stays that way, because it’s a good concept or would you say that it works even if the doctors are distributed?* *P: Nah no…I don’t think that’s good at all. I got all of them close by, the doctors, so I don’t have to drive far.* (Int10, lls. 602–603, 608–610, 630–633) P: Well, not that they said “go to the clinic”—I was asked... I1: Exactly and you think that’s good? *P: Yes. I think that is good.* (Int10, lls. 648–650)	Decentralized structures, doctors distributed in single clinics. The doctor takes the decisions without involving the patient or informal CG. Centralized structures such as polyclinics and medical centers. Shared decision making between doctor, patient and informal CG.	Yes
Assistance with daily activities	Grocery shopping, cleaning, getting dressed, showering, eating and drinking	*P: Well it has to! There is a bit of a must behind it… I don’t know what would be without them [mobile nursing service].* (Int8, lls. 226–227) *P: So the help from my wife is very important.* (Int9, lls. 256) *CG: Nope. No nursing service. They came before […], but they didn’t always come [at times we preferred] and then I said I’ll learn it and do it myself. […] because we are less bound to them like this, otherwise you are always bound to them. Because they don’t come when they want, but when they have time.* (Int1, lls. 117–119, 160–161)	Professional Family member	Yes
Characteristics of professional CG	*See sub-criteria.*	*P: […] The important thing is that you can deal with people, you are nice and friendly, you do the work that needs to be done. But I don’t need to study for that […] I think that’s nonsense. [...]* (Int10, lls. 474–476, 493–494) *P: Well I mean sure. I mean that they know what they are doing in their job, right?* *I1: Okay…so that’s important to you, the training and professional experience [of the nursing staff]?* *P: Well, I don’t have an overview of what they have to learn and don’t have to learn, but I mean if a nurse comes here [...] when I need help, I assume she knows how to help me.* *I1: And that is why training is important to you?* *P: Yes, that’s how I think about it. At the moment I don’t need it, but it can happen that I need it and then…* (Int2, lls. 203–210)	Empathy Education and work experience	Yes
Physical activities	Walks, gardening, sports, fishing, cleaning	*P: [Physical activity] I do for myself…* (Int5, lls. 110) *CG: Yes. Aren’t you doing group sports with your hands?* *I2: Exercise?* *P: Ooooooh yes! Then we sit there like that [hands up] and off we go. With the feet too!* (Int1, lls. 79–81) *I1: [...] the physical activity…so you said walking and gardening…but if you compare it to the social [activities], would it be important to you for your care that you have that [physical activities], or is it just the way it is?* *P: Yes, so that is important…that I can get out!* (Int7, lls. 127–130)	Alone Group	Yes
Dementia focused information and support for family CGs	Access to informational material via GP, Dementia support groups or the internet, self-help groups for informal CGs, inclusion in care decisions by professional CG and/or GP.	*I1: Is it important to you that your children […] are informed about your condition?* *P: Yes, my boy comes with me to the heart specialist…[…] with Dr. XXX… I always let them [children] come with me. I always say four ears hear more than two.* (Int10, lls. 543–546)	Difficult to receive Easy to receive Very easy to receive	Merged
Adjustments of the environment	Physical aids, homey adaptions of environment, assistive technology, sign-age, reduction of noise and clutter.	*P: Oh so for the apartment now [adjustments]?* *I1: Exactly.* *P: This is all fine here.* *I1: Have you preinstalled this here, for example handles in the shower to hold on to?* *P: Yes, everything preinstalled* *I1: Do you think that’s good?* *P: I think that’s good. But I don’t need it.* (Int7, lls. 420–427)	Not accessible In one room, e.g., the bathroom In the complete living area	No
Activities for sensory stimulation or relaxation	Music (e.g., listening, singing along, including in conversations and care), sensory stimulation with different materials, e.g., hand massage with lotion, smelling fresh flowers, preferably in a white and quiet room (refers to Snoezelen).	*I1: Do you like to touch this? Does that feel good?* *P: Well…* *I1: Or is that just part of life?* *P: Well…I haven’t given it that much thought yet…* (Int9, lls. 176–179) *I2: I’ll put it this way, it is just part of life.* *P: Yes, exactly…I mean when you’ve got some flowers…[…] of course you smell them. But is that so [something important]?* (Int2, lls. 147–149)	Difficult to access Activities to access outside home, e.g., in physiotherapy- and massage clinic Activities at home with physio therapist/masseur/music therapist	No
Attention and support with worries, feelings and memories	Telling life histories, work with reminiscence and self-validation.	*P: […] I don’t need that…* *I1: Don’t you have any worries?* *P: No, what should I worry about? [Shrugs shoulders]* (Int8, lls. 232–234) *P: No, here [day clinic] … I don’t have anyone I want to talk to about the problems. I’d rather be with a friend or something…but this, as I said, is intimate for me.* *I1: So with family, friends…?* *P: Hm.* (Int5, lls. 254–259)	Rarely available Accessible via a telephone hotline Through specifically educated advisor/priest/professional CG/family member	Merged
Waiting time	Possible additional waiting time, which would have to be taken into account for certain offers.	*P: Well, I mean…as a pensioner you have time and if you sit and wait for a quarter of an hour, that doesn’t matter.* (Int8, lls. 495–496) *P: […] People shouldn’t always complain right away anyway […] I don’t know any waiting time or almost not.* *I1: Ok. So you have had very good experiences?* *P: […] I mean […] I know how it works in a clinic. And I have no problem with that.* *I1: That means it doesn’t matter to you whether you wait a week or 14 days for an appointment.* *P: No.* *I1: And when you are at the doctor, you don’t care…* *P: It’s just the way it is.* (Int5, lls. 405–414)	11–14 days 7–10 days 3–6 days	No
Additional cost	Possible additional out-of-pocket payments.	*P: Important? It is there. That’s the way it is and if I want something, then I pay for it.* (Int4, lls. 427) *I1 […] Is this an issue for you or…* *P: No, not at all.* *I1: …is that how it is?* *P: I have a good pension and I can get by with it. [...] these are co-payments. There is nothing more to it.* (Int7, lls. 483–486, 496)	20 € per month (240 € per year) 40 € per month (480 € per year) 80 € per month (960 € per year)	No

Abbreviations: CG = Caregiver, GDR = German Democratic Republic, GP = General Practitioner, I1 = Interviewer 1, I2 = Interviewer 2, Int = Interview, P = Patient. _a_ As we realized during the interviews that the People living with Dementia most easily can understand and relate to the criteria by review of examples, we decided to delete extensive descriptions of the criteria as depicted in column one of Table 1 and only keep examples as lay terminology for the criteria.

**Table 4:** Results: Key quotations for categories 2, 4–6.

Category #	Key Quotations from Qualitative Data (Individual Interviews with *n* = 10 PlwD (and *n* = 3 Informal CGs))
(2) New patient relevant criteria of PCC	*I1: Is there anything, which was not included in the cards, but which you think we should write down? Because we also have blank cards and can create new criteria […] Is there anything we forgot?* *P: No.* *I1: It is well illustrated?* *P: It is well illustrated. * (Int3, lls. 289–295)
(4) Overlapping of criteria	*P: We find someone in the house to talk to. Sometimes, we sat outside on the bench. But that I [talk with other’s about my worries] well. Here with them [other residents in the apartment building]...I am the one who says “no, do this, do that”. [...]* *I1: Do you think that this [Criterion 6,Table 1] overlaps a bit or is the same as the social activities? Because you there [Criterion 1,Table 1] you also talk?* *P: Possible.* (Int8, lls. 238–245)
(5) Wording and comprehensibility	*P: Social aspects [Criterion 1,Table 1] means… [reads] that this will be and the other is in the future.* *I1: You don’t have to make it that complicated.* *P: No?* *I1: What is that for you? Do you have friends? Do you have a dog?* *P: I would have only thought about the medical side of this now. [...].* (Int5, lls. 13–17)
(6) Observations during interviews	
(a) Reactions by patient	*P: Let’s say the...how should you say this... what happens but...no...so...[participant is nervous] eh could you ask your question again briefly?* (Int5, lls 29–30) *I2: Um, this [criterion 8,Table 1] is about information and support if you have family members. […] you said you do everything by yourself, right? Hence, this might be a bit difficult to answer that [about criterion 8,Table 1]* *P: Family members…dementia. Yes, the dementia patients need us, they cannot be without us.* (Int6, lls 201–204) *P: And that they know [what to do], the nursing specialists [cf. criterion 7,Table 1] that is very clearly [important]. [...]. * (Int2, lls. 432)
(b) Interaction with informal CG	*P: What are you doing now? [towards informal CG]* *CG: No! That we both can [do something]. That you are with me is of no use to you either, you have to be with people who are just as sick as you!* *P: Yes yes, hm.* *CG: They talk to each other very differently…* *P: Well if you want to deport me…* (Int1, lls. 309–314) *P: Yes, I need a change. I need it...very often...anyways, my wife is an impediment with regard to this question because she is afraid that I will somehow tip over or something. But personally...* *CG: Well, I’m always afraid that he will fall there, because it is not flat in the garden. And he fell there a few times. And then I’m afraid that he will fall again. And that’s why I only let him do things where the danger of falling is not likely. Where he doesn’t have to bend down, where he can stand up straight.* (Int9, lls. 207–212) *I1: Ok. Great. You’re doing really well. That helps us a lot. So activities of daily living. It’s like eating, showering, everything you do every day. Getting dressed...I think you are still very physically fit. You can still do it all [by yourself].* *P: Yeah.* *I1: Do you currently need help with [anything] or do you do everything on your own?* *P: I do a lot of things on my own. I don’t want to say everything, but a lot.* *I2: Most of it, yes?* *P: Yes.* *I1: If you should ever need help, would it be important for you to get help?* *P: Yeah. Well. I have a wife who knows everything. She also studied.* (Int4, lls. 235–244)
(c) Explorative vs. card game	*I1: We thought that [social] activities for example could be individual or group discussions, writing letters, videos, working with figures. But you cannot relate anything to that?* *P: No. Why should we waste our time with this? * (Int1, lls. 25–27) *I1: Okay. So you would say that this [criterion 10,Table 1] is maybe of middle importance?* *P: Yeah well...not at the moment, as long as I can still do it by myself. But if I then...so if I were to forget that...then...[…] We chose Dr. [XXX]. We didn’t know her...but she was nearby. So we didn’t have to walk far. Or take the bus or something.* *I1: Okay. So. Proximity is important...it’s kind of important. [...].* (Int2, lls. 303–310) *I1: How would that be, should you ever need that [adjustments of the environment]? Would you like to have that then?* *P: I think I can take my time. Doesn’t have to be now... from now on I’m sick and now I have to [get help]…* (Int5, lls. 347–349)
(d) Context	*I1: Exactly. [laughs] And there used to be polyclinics in the GDR.* *P: Yes.* *I1: How do you like that, the concept?* *P: Very good! In general I find everything related to GDR very good.* (Int4, lls. 391–394)
(e) COVID-19	*P: Well, what can I think of [ad criterion 1,Table 1]—well now, due to Corona, drinking coffee has been cancelled. Otherwise, we always had 1–2 h of drinking coffee together [in the community housing clubroom] on Wednesdays and Thursdays in the afternoons. * (Int2, lls. 15–16) *I1: And do you think it’s good that you can do something like that…go for a walk and something?* *P: Yeah well, I have to like it. I can no longer travel, I imagined my retirement to be different. But everything is gone and now the disease [is there] too. The big one.* *I1: You mean Corona?* *P: Corona, that’s exactly what I mean. I always forget the name.* (Int7, lls. 101–105) *P: Yes, but this is no longer…otherwise you would have had more contact [sad].* *I1: Hm. Because of Corona it is no longer [the contact]?* *P: Yes.* *CG: Yes, unfortunately it is...really bad with Corona.* (Int9, lls. 39–42) *CG: We did sports until Corona. We’re still in the sports group, but we’ll cancel our membership because he can’t do it anymore. He can no longer participate, no matter what we did there. It doesn’t work anymore. He has lost so much lately.* (Int9, lls. 83–85)

Abbreviations: CG = Caregiver, GDR = German Democratic Republic, I1 = Interviewer 1, I2 = Interviewer 2, Int = Interview, lls = lines, P = Patient, PCC = Person-Centered Care.

## Data Availability

Data and methods used are presented in sufficient detail in the paper so that other researcher can replicate the work. Raw data will not be made publicly available to protect patient confidentiality.

## Supplementary Materials

The following supporting information can be downloaded at: https://www.mdpi.com/article/10.3390/ijerph19137629/s1, Supplemental Codebook S1.

## References

[1] Prince M., Comas-Herrera A., Knapp M., Guerchet M., Karagiannidou M. (2016). World Alzheimer Report 2016—Improving Healthcare for People Living with Dementia: Coverage, Quality and Costs Now and in the Future.

[2] World Health Organization Dementia Fact Sheet. https://www.who.int/news-room/fact-sheets/detail/dementia.

[3] Vos T., Lim S.S., Abbafati C., Abbas K.M., Abbasi M., Abbasifard M., Abbasi-Kangevari M., Abbastabar H., Abd-Allah F., Abdelalim A. (2020). Global burden of 369 diseases and injuries in 204 countries and territories, 1990–2019: A systematic analysis for the Global Burden of Disease Study 2019. Lancet.

[4] Prince M., Bryce R., Ferri C. (2011). World Alzheimer Report 2011—The Benefits of Early Diagnosis and Intervention.

[5] Alzheimer’s Association Dementia Care Practice Recommendations. https://www.alz.org/professionals/professional-providers/dementia_care_practice_recommendations.

[6] National Institute for Health and Care Excellence (2018). Dementia: Assessment, Management and Support for People Living with Dementia and Their Carers (NG97). NICE Guideline.

[7] The National Board of Health and Welfare (2017). Nationella Riktlinjer för Vård Och Omsorg Vid Demenssjukdom. Stöd för Styrning Och Ledning.

[8] NHMRC Partnership Centre for Dealing with Cognitive and Related Functional Decline in Older People (2016). Clinical Practice Guidelines and Principles of Care for People with Dementia.

[9] Dely H., Verschraegen J., Setyaert J. (2018). You and Me, Together We Are Human—A Reference Framework for Quality of Life, Housing and Care for People with Dementia.

[10] Savaskan E., Bopp-Kistler I., Buerge M., Fischlin R., Georgescu D., Giardini U., Hatzinger M., Hemmeter U., Justiniano I., Kressig R.W. (2014). Empfehlungen zur Diagnostik und Therapie der Behavioralen und Psychologischen Symptome der Demenz (BPSD). Praxis.

[11] Danish Health Authority (2019). Forebyggelse og Behandling af Adfærdsmæssige og Psykiske Symptomer hos Personer Med Demens. National Klinisk Retningslinje.

[12] Norwegian Ministry of Health and Care Services (2015). Dementia Plan 2020—A More Dementia-Friendly Society.

[13] Morgan S., Yoder L. (2012). A concept analysis of person-centered care. J. Holist. Nurs..

[14] Kitwood T., Bredin K. (1992). Towards a theory of dementia care: Personhood and well-being. Ageing Soc..

[15] Kitwood T. (1997). Dementia Reconsidered: The Person Comes First (Rethinking Ageing Series).

[16] Lepper S., Rädke A., Wehrmann H., Michalowsky B., Hoffmann W. (2020). Preferences of Cognitively Impaired Patients and Patients Living with Dementia: A Systematic Review of Quantitative Patient Preference Studies. J. Alzheimer’s Dis..

[17] Wehrmann H., Michalowsky B., Lepper S., Mohr W., Raedke A., Hoffmann W. (2021). Priorities and Preferences of People Living with Dementia or Cognitive Impairment—A Systematic Review. Patient Prefer. Adherence.

[18] Ho M.H., Chang H.R., Liu M.F., Chien H.W., Tang L.Y., Chan S.Y., Liu S.H., John S., Traynor V. (2021). Decision-Making in People With Dementia or Mild Cognitive Impairment: A Narrative Review of Decision-Making Tools. J. Am. Med. Dir. Assoc..

[19] Harrison Dening K., King M., Jones L., Vickerstaff V., Sampson E.L. (2016). Correction: Advance Care Planning in Dementia: Do Family Carers Know the Treatment Preferences of People with Early Dementia?. PLoS ONE.

[20] Van Haitsma K., Curyto K., Spector A., Towsley G., Kleban M., Carpenter B., Ruckdeschel K., Feldman P.H., Koren M.J. (2013). The preferences for everyday living inventory: Scale development and description of psychosocial preferences responses in community-dwelling elders. Gerontologist.

[21] Mühlbacher A. (2017). Ohne Patientenpräferenzen kein sinnvoller Wettbewerb. Dtsch. Ärzteblatt.

[22] Groenewoud S., Van Exel N.J.A., Bobinac A., Berg M., Huijsman R., Stolk E.A. (2015). What influences patients’ decisions when choosing a health care provider? Measuring preferences of patients with knee arthrosis, chronic depression, or Alzheimer’s disease, using discrete choice experiments. Health Serv. Res..

[23] Mühlbacher A.C., Kaczynski A., Zweifel P., Johnson F.R. (2016). Experimental measurement of preferences in health and healthcare using best-worst scaling: An overview. Health Econ. Rev..

[24] Mühlbacher A.C., Kaczynski A. (2013). Der Analytic Hierarchy Process (AHP): Eine Methode zur Entscheidungsunterstützung im Gesundheitswesen. Pharm. Ger. Res. Artic..

[25] Danner M., Vennedey V., Hiligsmann M., Fauser S., Gross C., Stock S. (2016). How Well Can Analytic Hierarchy Process be Used to Elicit Individual Preferences? Insights from a Survey in Patients Suffering from Age-Related Macular Degeneration. Patient.

[26] Thokala P., Devlin N., Marsh K., Baltussen R., Boysen M., Kalo Z., Longrenn T., Mussen F., Peacock S., Watkins J. (2016). Multiple Criteria Decision Analysis for Health Care Decision Making—An Introduction: Report 1 of the ISPOR MCDA Emerging Good Practices Task Force. Value Health.

[27] Marsh K., IJzerman M., Thokala P., Baltussen R., Boysen M., Kaló Z., Lönngren T., Mussen F., Peacock S., Watkins J. (2016). Multiple Criteria Decision Analysis for Health Care Decision Making—Emerging Good Practices: Report 2 of the ISPOR MCDA Emerging Good Practices Task Force. Value Health.

[28] Abiiro G.A., Leppert G., Mbera G.B., Robyn P.J., De Allegri M. (2014). Developing attributes and attribute-levels for a discrete choice experiment on micro health insurance in rural Malawi. BMC Health Serv. Res..

[29] Hollin I.L., Craig B.M., Coast J., Beusterien K., Vass C., DiSantostefano R., Peay H. (2020). Reporting formative qualitative research to support the development of quantitative preference study protocols and corresponding survey instruments: Guidelines for authors and reviewers. Patient-Patient-Cent. Outcomes Res..

[30] Coast J., Al-Janabi H., Sutton E.J., Horrocks S.A., Vosper A.J., Swancutt D.R., Flynn T.N. (2012). Using qualitative methods for attribute development for discrete choice experiments: Issues and recommendations. Health Econ..

[31] Creswell J.W., Poth C.N. (2016). Qualitative Inquiry and Research Design: Choosing among Five Approaches.

[32] Robson C. (2002). Real World Research: A Resource for Social Scientists and Practitioner-Researchers.

[33] German Center for Neurodegenerative Diseases e.V. (DZNE) PreDemCare: Moving towards Person-Centered Care of People with Dementia—Elicitation of Patient and Physician Preferences for Care. https://www.dzne.de/en/research/studies/projects/predemcare/.

[34] Rädke A., Mohr W., Michalowsky B., Hoffmann W. (2022). POSA422 Preferences for Person-Centred Care Among People with Dementia in Comparison to Physician’s Judgments: Study Protocol for the Predemcare Study. Value Health.

[35] Creswell J.W., Clark V.L.P. (2017). Designing and Conducting Mixed Methods Research.

[36] Mohr W., Rädke A., Afi A., Edvardsson D., Mühlichen F., Platen M., Roes M., Michalowsky B., Hoffmann W. (2021). Key Intervention Categories to Provide Person-Centered Dementia Care: A Systematic Review of Person-Centered Interventions. J. Alzheimer’s Dis. JAD.

[37] Saaty T.L. (2003). Decision-making with the AHP: Why is the principal eigenvector necessary. Eur. J. Oper. Res..

[38] Manthey L. (2007). Methoden der Präferenzmessung: Grundlagen, Konzepte und Experimentelle Untersuchungen.

[39] Eichler T., Thyrian J.R., Dreier A., Wucherer D., Köhler L., Fiß T., Böwing G., Michalowsky B., Hoffmann W. (2014). Dementia care management: Going new ways in ambulant dementia care within a GP-based randomized controlled intervention trial. Int. Psychogeriatr..

[40] Mühlbacher A.C., Kaczynski A. (2016). Making good decisions in healthcare with multi-criteria decision analysis: The use, current research and future development of MCDA. Appl. Health Econ. Health Policy.

[41] Kuruoglu E., Guldal D., Mevsim V., Gunvar T. (2015). Which family physician should I choose? The analytic hierarchy process approach for ranking of criteria in the selection of a family physician. BMC Med. Inform. Decis. Mak..

[42] Bamberger M., Mabry L. (2019). RealWorld Evaluation: Working under Budget, Time, Data, and Political Constraints.

[43] Green J., Thorogood N. (2018). Qualitative Methods for Health Research.

[44] Dickson K., Lafortune L., Kavanagh J., Thomas J., Mays N., Erens B. (2012). Non-Drug Treatments for Symptoms in Dementia: An Overview of Systematic Reviews of Non-Pharmacological Interventions in the Management of Neuropsychiatric Symptoms and Challenging Behaviours in Patients with Dementia.

[45] Clarkson P., Hughes J., Xie C., Larbey M., Roe B., Giebel C.M., Jolley D., Challis D., Group H.D.P.M. (2017). Overview of systematic reviews: Effective home support in dementia care, components and impacts—Stage 1, psychosocial interventions for dementia. J. Adv. Nurs..

[46] Chester H., Clarkson P., Davies L., Sutcliffe C., Davies S., Feast A., Hughes J., Challis D., Members of the HOST-D (Home Support in Dementia) Programme Management Group (2018). People with dementia and carer preferences for home support services in early-stage dementia. Aging Ment. Health.

[47] Ballard C., Corbett A., Orrell M., Williams G., Moniz-Cook E., Romeo R., Woods B., Garrod L., Testad I., Woodward-Carlton B. (2018). Impact of person-centred care training and person-centred activities on quality of life, agitation, and antipsychotic use in people with dementia living in nursing homes: A cluster-randomised controlled trial. PLoS Med..

[48] Boersma P., van Weert J.C., Lissenberg-Witte B.I., van Meijel B., Dröes R.-M. (2019). Testing the implementation of the Veder Contact Method: A theatre-based communication method in dementia care. Gerontologist.

[49] Cohen-Mansfield J., Thein K., Marx M.S., Dakheel-Ali M., Freedman L. (2012). Efficacy of nonpharmacologic interventions for agitation in advanced dementia: A randomized, placebo-controlled trial. J. Clin. Psychiatry.

[50] Fossey J., Ballard C., Juszczak E., James I., Alder N., Jacoby R., Howard R. (2006). Effect of enhanced psychosocial care on antipsychotic use in nursing home residents with severe dementia: Cluster randomised trial. BMJ.

[51] Lawton M.P., Van Haitsma K., Klapper J., Kleban M.H., Katz I.R., Corn J. (1998). A stimulation-retreat special care unit for elders with dementing illness. Int. Psychogeriatr..

[52] Tay F.H.E., Thompson C.L., Nieh C.M., Nieh C.C., Koh H.M., Tan J.J.C., Yap P.L.K. (2018). Person-centered care for older people with dementia in the acute hospital. Alzheimer Dement. Transl. Res. Clin. Interv..

[53] van der Ploeg E.S., Eppingstall B., Camp C.J., Runci S.J., Taffe J., O’Connor D.W. (2013). A randomized crossover trial to study the effect of personalized, one-to-one interaction using Montessori-based activities on agitation, affect, and engagement in nursing home residents with Dementia. Int. Psychogeriatr..

[54] Van Haitsma K.S., Curyto K., Abbott K.M., Towsley G.L., Spector A., Kleban M. (2015). A randomized controlled trial for an individualized positive psychosocial intervention for the affective and behavioral symptoms of dementia in nursing home residents. J. Gerontol. Ser. B Psychol. Sci. Soc. Sci..

[55] Verbeek H., Zwakhalen S.M., van Rossum E., Ambergen T., Kempen G.I., Hamers J.P. (2014). Effects of small-scale, home-like facilities in dementia care on residents’ behavior, and use of physical restraints and psychotropic drugs: A quasi-experimental study. Int. Psychogeriatr..

[56] van Weert J.C., van Dulmen A.M., Spreeuwenberg P.M., Ribbe M.W., Bensing J.M. (2005). Effects of snoezelen, integrated in 24 h dementia care, on nurse–patient communication during morning care. Patient Educ. Couns..

[57] Sloane P.D., Hoeffer B., Mitchell C.M., McKenzie D.A., Barrick A.L., Rader J., Stewart B.J., Talerico K.A., Rasin J.H., Zink R.C. (2004). Effect of person-centered showering and the towel bath on bathing-associated aggression, agitation, and discomfort in nursing home residents with dementia: A randomized, controlled trial. J. Am. Geriatr. Soc..

[58] Chenoweth L., King M.T., Jeon Y.-H., Brodaty H., Stein-Parbury J., Norman R., Haas M., Luscombe G. (2009). Caring for Aged Dementia Care Resident Study (CADRES) of person-centred care, dementia-care mapping, and usual care in dementia: A cluster-randomised trial. Lancet Neurol..

[59] Eritz H., Hadjistavropoulos T., Williams J., Kroeker K., Martin R.R., Lix L.M., Hunter P.V. (2016). A life history intervention for individuals with dementia: A randomised controlled trial examining nursing staff empathy, perceived patient personhood and aggressive behaviours. Ageing Soc..

[60] Rokstad A.M.M., Røsvik J., Kirkevold Ø., Selbaek G., Benth J.S., Engedal K. (2013). The effect of person-centred dementia care to prevent agitation and other neuropsychiatric symptoms and enhance quality of life in nursing home patients: A 10-month randomized controlled trial. Dement. Geriatr. Cogn. Disord..

[61] Testad I., Mekki T.E., Førland O., Øye C., Tveit E.M., Jacobsen F., Kirkevold Ø. (2016). Modeling and evaluating evidence-based continuing education program in nursing home dementia care (MEDCED)—training of care home staff to reduce use of restraint in care home residents with dementia. A cluster randomized controlled trial. Int. J. Geriatr. Psychiatry.

[62] Van Bogaert P., Tolson D., Eerlingen R., Carvers D., Wouters K., Paque K., Timmermans O., Dilles T., Engelborghs S. (2016). SolCos model-based individual reminiscence for older adults with mild to moderate dementia in nursing homes: A randomized controlled intervention study. J. Psychiatr. Ment. Health Nurs..

[63] Chenoweth L., Forbes I., Fleming R., King M., Stein-Parbury J., Luscombe G., Kenny P., Jeon Y.-H., Haas M., Brodaty H. (2014). PerCEN: A cluster randomized controlled trial of person-centered residential care and environment for people with dementia. Int. Psychogeriatr..

[64] van de Ven G., Draskovic I., Adang E.M., Donders R., Zuidema S.U., Koopmans R.T., Vernooij-Dassen M.J. (2013). Effects of dementia-care mapping on residents and staff of care homes: A pragmatic cluster-randomised controlled trial. PLoS ONE.

[65] Villar F., Celdrán M., Vila-Miravent J., Fernández E. (2019). Involving institutionalized people with dementia in their care-planning meetings: Impact on their quality of life measured by a proxy method: Innovative Practice. Dementia.

[66] Drummond M.F., Sculpher M.J., Claxton K., Stoddart G.L., Torrance G.W. (2015). Methods for the Economic Evaluation of Health Care Programmes.

[67] Mühlbacher A.C., Rudolph I., Lincke H.-J., Nübling M. (2009). Preferences for treatment of attention-deficit/hyperactivity disorder (ADHD): A discrete choice experiment. BMC Health Serv. Res..

[68] Mühlbacher A.C., Bethge S. (2015). Patients’ preferences: A discrete-choice experiment for treatment of non-small-cell lung cancer. Eur. J. Health Econ..

[69] Mühlbacher A.C., Kaczynski A., Dippel F.-W., Bethge S. (2018). Patient priorities for treatment attributes in adjunctive drug therapy of severe hypercholesterolemia in germany: An analytic hierarchy process. Int. J. Technol. Assess. Health Care.

[70] Mühlbacher A., Bethge S., Kaczynski A., Juhnke C. (2015). Objective criteria in the medicinal therapy for type II diabetes: An analysis of the patients’ perspective with analytic hierarchy process and best-worst scaling. Gesundh. (Bundesverb. Der Arzte Des. Offentlichen Gesundh.).

[71] Mühlbacher A.C., Kaczynski A. (2016). The expert perspective in treatment of functional gastrointestinal conditions: A multi-criteria decision analysis using AHP and BWS. J. Multi-Criteria Decis. Anal..

[72] Weernink M.G., van Til J.A., Groothuis-Oudshoorn C.G., IJzerman M.J. (2017). Patient and public preferences for treatment attributes in Parkinson’s disease. Patient-Patient-Cent. Outcomes Res..

[73] Danner M., Vennedey V., Hiligsmann M., Fauser S., Stock S. (2016). Focus Groups in Elderly Ophthalmologic Patients: Setting the Stage for Quantitative Preference Elicitation. Patient.

[74] Folstein M.F., Folstein S.E., McHugh P.R. (1975). “Mini-mental state”. A practical method for grading the cognitive state of patients for the clinician. J. Psychiatr. Res..

[75] Zaudig M., Mittelhammer J., Hiller W., Pauls A., Thora C., Morinigo A., Mombour W. (1991). SIDAM—A structured interview for the diagnosis of dementia of the Alzheimer type, multi-infarct dementia and dementias of other aetiology according to ICD-10 and DSM-III-R. Psychol. Med..

[76] Mayring P. Qualitative Content Analysis: Theoretical Foundation, Basic Procedures and Software Solution. http://nbn-resolving.de/urn:nbn:de:0168-ssoar-395173.

[77] Mayring P. Qualitative Content Analysis. https://www.qualitative-research.net/index.php/%20fqs/article/view/1089/2385.

[78] Hsieh H.-F., Shannon S.E. (2005). Three Approaches to Qualitative Content Analysis. Qual. Health Res..

[79] Xanthopoulou P., McCabe R. (2019). Subjective experiences of cognitive decline and receiving a diagnosis of dementia: Qualitative interviews with people recently diagnosed in memory clinics in the UK. BMJ Open.

[80] Bacsu J.R., O’Connell M.E., Webster C., Poole L., Wighton M.B., Sivananthan S. (2021). A scoping review of COVID-19 experiences of people living with dementia. Can. J. Public Health.

[81] Michalowsky B., Hoffmann W., Bohlken J., Kostev K. (2020). Effect of the COVID-19 lockdown on disease recognition and utilisation of healthcare services in the older population in Germany: A cross-sectional study. Age Ageing.

[82] Van Haitsma K., Abbott K.M., Arbogast A., Bangerter L.R., Heid A.R., Behrens L.L., Madrigal C. (2020). A Preference-Based Model of Care: An Integrative Theoretical Model of the Role of Preferences in Person-Centered Care. Gerontologist.

[83] van Til J.A., Ijzerman M.J. (2014). Why Should Regulators Consider Using Patient Preferences in Benefit-risk Assessment?. PharmacoEconomics.

[84] Edvardsson D., Varrailhon P., Edvardsson K. (2014). Promoting person-centeredness in long-term care: An exploratory study. J. Gerontol. Nurs..

[85] Fazio S., Pace D., Flinner J., Kallmyer B. (2018). The Fundamentals of Person-Centered Care for Individuals With Dementia. Gerontologist.

[86] Jayadevappa R., Chhatre S., Gallo J.J., Wittink M., Morales K.H., Lee D.I., Guzzo T.J., Vapiwala N., Wong Y.-N., Newman D.K. (2019). Patient-centered preference assessment to improve satisfaction with care among patients with localized prostate cancer: A randomized controlled trial. J. Clin. Oncol..

[87] Bridges J.F.P., Hauber A.B., Marshall D., Lloyd A., Prosser L.A., Regier D.A., Johnson F.R., Mauskopf J. (2011). Conjoint Analysis Applications in Health—a Checklist: A Report of the ISPOR Good Research Practices for Conjoint Analysis Task Force. Value Health.

[88] Kløjgaard M.E., Bech M., Søgaard R. (2012). Designing a stated choice experiment: The value of a qualitative process. J. Choice Model..

[89] Hannemann N., Götz N.-A., Schmidt L., Hübner U., Babitsch B. (2021). Patient connectivity with healthcare professionals and health insurer using digital health technologies during the COVID-19 pandemic: A German cross-sectional study. BMC Med. Inform. Decis. Mak..

[90] Thyrian J.R., Fiß T., Dreier A., Böwing G., Angelow A., Lueke S., Teipel S., Fleßa S., Grabe H.J., Freyberger H.J. (2012). Life-and person-centred help in Mecklenburg-Western Pomerania, Germany (DelpHi): Study protocol for a randomised controlled trial. Trials.

[91] Given L.M. (2008). The SAGE Encyclopedia of Qualitative Research Methods.

[92] Beuscher L., Grando V.T. (2009). Challenges in conducting qualitative research with individuals with dementia. Res. Gerontol. Nurs..

[93] Neidhardt K., Wasmuth T. (2012). Die Gewichtung Multipler Patientenrelevanter Endpunkte—Ein Methodischer Vergleich von Conjoint Analyse und Analytic Hierarchy Process unter Berücksichtigung des Effizienzgrenzenkonzepts des IQWIG.

[94] Graneheim U.H., Lundman B. (2004). Qualitative content analysis in nursing research: Concepts, procedures and measures to achieve trustworthiness. Nurse Educ. Today.

