# Supplementary material for: Development of a Quantitative Instrument to Elicit Patient Preferences for Person-Centered Dementia Care Stage 1: A Formative Qualitative Study to Identify Patient Relevant Criteria for Experimental Design of an Analytic Hierarchy Process
Source: Int J Environ Res Public Health. 2022 Jun 22;19(13):7629. doi: 10.3390/ijerph19137629 (PMC9266267; doi:10.3390/ijerph19137629)
Supplement: Supplementary file 1 [file ijerph-19-07629-s001.zip › ijerph-1663615-supplementary.pdf]

Supplemental 1 – Codebook

1. Importance of criteria, i.e. identification of actually patient-relevant criteria of Person-Centered Care (PCC)

Brief Definition: Refers to the indication of importance of criteria portrayed on cards. The main source of information are pictures with results from ranking games. Information from transcript in explorative card game and field notes only serve as additional information to have an indicator for consistency of preferences. The main goal is to reduce the amount of criteria to a feasible number that entail the most patient-relevant criteria for the later pairwise-comparisons-based Analytic Hierarchy Process (AHP)-survey.

Inclusion criteria: Apply this to all meaning units (i.e. text passages) that describe the perceived and expressed importance of criteria as expressed by the patient.

Exclusion Criteria: Do not use this for meaning units that indicate new criteria (cf. 2.), potential levels / sub-criteria (cf. 3.), overlapping of criteria and/or sub-criteria (cf. 4.), wording (and appropriateness of icons for sub-criteria) (cf. 5.), or other new inductive categories that might arise from the transcript (cf. 6.).

Integration with subsequent categories: The identification of the most important, i.e. patient-relevant criteria will influence the remaining categories, as it will provide information for the first level of the AHP-hierarchy. In some places, it might rather be appropriate to merge overlapping criteria than to delete criteria from the list.

Example: *(P_Int2_202): "I mean of course. I mean [of course it is important] that they know what they are doing in their job, right [Criterion 7]?!"*

2. Indication of *new* patient-relevant criteria of PCC

Brief definition: Refers to potentially new and unknown patient-relevant criteria of PCC that might arise from the interview, especially from the explorative part, and would be noted at additional empty cards carried by the interviewers. The patients were explicitly asked for any potential criteria missed after the first explorative card game.

Inclusion criteria: Apply this to all meaning units that describe new patient-relevant criteria that were not identified by the previous systematic literature review and are mentioned to be important to the patient(s). This might also arise from the context of the explorative part without an explicit indication by the patient.

Exclusion Criteria: Do not use this for meaning units that indicate importance of already existing criteria (cf. 1.), potential levels / sub-criteria (cf. 3.), overlapping of criteria and/or sub-criteria (cf. 4.),

wording (and appropriateness of icons for sub-criteria) (cf. 5.), or other new inductive categories that might arise from the transcript (cf. 6.).

Integration with other categories: The identification of potentially new and unknown patient-relevant criteria of PCC will influence the remaining categories, as it will provide information for potential new criteria on the first level of the AHP-hierarchy. This might seem contrary to the aim to reduce the number of criteria identified via the previous systematic literature review to a feasible number of criteria. However, the criteria need to mirror what is actually patient-relevant. Hence, potential new criteria might replace other previous criteria.

Example: *(P_Int4_538): "Well, what is important are the activities that you need in daily life and that are there or that you should do."*

3. Indication of potential sub-criteria

Brief definition: Refers to potential sub-criteria that form the third level in the AHP-hierarchy and describe the characteristics or manifestations of the criteria of PCC. Important is a "realistic portrayal" of the actual situation, i.e. how the patients actually (have) experience(d) the manifestations of the different criteria in reality.

Inclusion criteria: Apply this to all meaning units that indicate or describe patient-relevant and realistic sub-criteria. Information about this might arise from elaborations by the patients in the explorative part. If time is sufficient, the interviewers had prepared additional cards with potential sub-criteria and respective icons to show to the patient and ask about appropriateness. Feasibility of this last card game was however highly situation dependent, most and foremost dependent on both physical and cognitive capacities of the patient at the day of the interview.

Exclusion Criteria: Do not use this for meaning units that indicate importance of already existing criteria (cf. 1.), potential new patient-relevant criteria (cf. 2.), overlapping of criteria and/or sub-criteria (cf. 4.), wording (and appropriateness of icons for sub-criteria) (cf. 5.), or other new inductive categories that might arise from the transcript (cf. 6.).

Example: *(CG_Int1_143-145): "So contacts with others yes [ad Attribute 1 or 6], but not with particular people like a priest or so no. Okay..if she would come and talk to him, yes. So when my sister or my son come and talk to him. That he likes."*

4. Overlapping of criteria (and sub-criteria)

Brief description: Refers to indicators of potential overlapping of criteria (and sub-criteria).

Inclusion criteria: Apply this to all meaning units that give an indication of potential overlapping of criteria (and sub-criteria). This might mostly arise from the explorative part from the patients

elaborations about the criteria they are presented with. However, indication of overlapping can also arise during card games two and three and during the ranking of criteria, when patients hesitate to make a choice and underline this with elaborations that indicate overlapping.

Exclusion criteria: Do not use this for meaning units that indicate importance of already existing criteria (cf. 1.), potential new patient-relevant criteria (cf. 2.), indication of potential sub-criteria (cf. 3.), wording (and appropriateness of icons for sub-criteria) (cf. 5.), or other new inductive categories that might arise from the transcript (cf. 6.).

Example: *(P_Int2_28): "Yes…an hour or so is that always, chair sports [group activity, people sitting at chairs in a circle and practicing certain physical exercises]. But as I said…then you are at least with people."*

5. Wording

Brief description: Refers to indications by patients about incomprehensibility of wording for criteria (and sub-criteria) titles and descriptions, as well as inappropriateness of graphical design for icons related to sub-criteria. Also applies to words that might be perceived as upsetting, such as "dementia", "memories", by those PwD that find it hard to cope with the dementia diagnosis. Furthermore, this applies to concrete examples the PwD relate the criteria (and sub-critera) to.

Inclusion criteria: Apply this to all meaning units that give an indication of incomprehensibility of text and/or icons and inappropriateness of icons and/or text.

Exclusion criteria: Do not use this for meaning units that indicate importance of already existing criteria (cf. 1.), potential new patient-relevant criteria (cf. 2.), indication of potential sub-criteria (cf. 3.), overlapping of criteria (and sub-criteria) (cf. 4.), or other new inductive categories that might arise from the transcript (cf. 6.).

Example: *(P_Int4_256_288-289): "Towel bath? What is that? […] Memories. Memories also arise during conversation – oh God yes. […]"*

6. Other observations

Brief description: Refers to categories that arise from the context of the transcribed material, i.e. reactions by the patient, interactions with caregiver (if attending), reactions to the card game, the both broad and narrow context the interview was conducted in, reactions to interviewers.

Inclusion criteria: Apply this to all meaning units that give an indication of incomprehensibility of text and/or icons and inappropriateness of icons and/or text.

Exclusion criteria: Do not use this for meaning units that indicate importance of already existing criteria (cf. 1.), potential new patient-relevant criteria (cf. 2.), indication of potential sub-criteria (cf. 3.),

overlapping of criteria (and sub-criteria) (cf. 4.), or wording (and appropriateness of icons for sub-criteria) (cf. 5.).

Examples:

a. **Reactions by patient**
    i. Study participants want to “perform well”
    ii. Difficulties to deal with the disease “dementia”
    iii. Social-desirability during answers (attendance of informal and professional CGs)
b. **Interaction with informal caregiver**
    i. Anxiety to loose caregiver, wants to be liked
    ii. Caregiver is more fit and can be encroaching – accepted by patient
    iii. Informal CG is expected to perform care tasks
c. **Explorative vs. ranking card game**
    i. Different depending on dementia stage
    ii. “Proxy”-rating, i.e. „imagine if…“ – thoughts about an unknown future
    iii. Inconsistent answers / expressions of preferences
d. **Context**
    i. Region: former German Democratic Republic (GDR)
e. **Corona**
